# Advanced Analysis of the Properties of Solid-Wire Electric Contacts Produced by Ultrasonic Welding and Soldering

**DOI:** 10.3390/ma17020334

**Published:** 2024-01-09

**Authors:** Andraž Logar, Damjan Klobčar, Aleš Nagode, Uroš Trdan, Gregor Černivec, Abhay Sharma

**Affiliations:** 1 Bosch Rexroth d.o.o., Zgornji Brnik 410, 4210 Brnik, Slovenia; andraz.logar@boschrexroth.si (A.L.); gregor.cernivec@boschrexroth.si (G.Č.); 2 Laboratory for Welding, Faculty of Mechanical Engineering, University of Ljubljana, Aškerčeva 6, 1000 Ljubljana, Slovenia; uros.trdan@fs.uni-lj.si; 3 Department of Materials and Metallurgy, University of Ljubljana, Aškerčeva 12, 1000 Ljubljana, Slovenia; ales.nagode@ntf.uni-lj.si; 4 Faculty of Engineering Technology, KU Leuven, Jan Pieter de Nayerlaan 5, 2860 Sint-Katelijne Waver, Belgium; abhay.sharma@kuleuven.be

**Keywords:** solid copper wires, ultrasonic welding, soldering, thermal shock test, joint strength, electrical conductivity

## Abstract

The current article presents an advanced analysis of the properties of solid-wire electric contacts produced with ultrasonic welding and soldering. Soldering is generally used to join thin, solid copper wires to produce electrical contacts in small-volume production, as ultrasonic welding does not provide acceptable peel force and tensile strength due to the deformation and thinning of the wires. In this article, ultrasonic welding of thin, solid copper wires using a ring before and after a thermal shock test is discussed and compared with the standard soldering technique. The thermal shock test was carried out in the temperature range from −30 to 150 °C. Half of the samples, for both the joining techniques and the wires, were subjected to the thermal shock test; the other half were not. Investigations included electrical resistance tests, optical and SEM microscopy, XRD, microhardness measurements, peel tests, tensile tests, and fractographic analysis. The electrical resistance test, microscopy, microhardness measurements, and fracture examinations showed no differences between the thermal shock-exposed and the non-exposed samples with the same joining process. In mechanical tests, the ultrasonic joint demonstrated superior strength compared to the soldered joint.

## 1. Introduction

Electric motors are increasingly present in our everyday life, powering appliances, smart homes, buildings, and electric mobility. In high-power motors, copper wires are the most efficient choice due to their electrical resistance, energy efficiency, and heat management. For these wires to be effectively used, they need to be connected with suitable electrical contacts that yield mechanical strength, resistance to vibrations, resistance to temperature changes, and high electrical conductivity. Engineers must select appropriate joining technologies, considering joint loading conditions, lifetime, and manufacturability. Each joining process has certain advantages and disadvantages that should be carefully considered when choosing the optimal joining technology for solid wires, conductors, or braids. This is even more important when the joining of dissimilar materials is needed.

Commonly used technologies for connecting wires and conductors include electrical resistance welding, soldering, crimping, or welding with mechanical energy [1]. Electrical resistance welding processes utilize Joule heat for joining, such as in (i) hot crimping, where the process allows simultaneous removal of varnish and welding; (ii) compaction, where the joint is formed together with Joule heating and deformation; (iii) the thermode process, where a heated tungsten electrode melts the varnish in the beginning and then welds the wires together; and (iv) resistance soldering, where fluxes, solders, and resistance heating are/is used for soldering [1]. While these processes allow the joining of varnished wires, they have limitations, such as requiring different electrodes and welding parameters for wires with different cross-sections, thus limiting their use in low-series production.

Soldering, which employs various heating technologies, like oxy-fuel, electrical resistance, induction, and laser [2], offers universality and allows the connection of wires and conductors with different cross-sections without process changes. The quality of a soldered joint depends on the optimal joint gap, joint overlap, joint cleanliness, and homogenous temperature throughout the joint and the stability of influencing factors. No reduction in the wire cross-section results in a larger joint, thus causing low electrical resistance and high joint strength. Because the filler material and the base material have different chemical compositions, such joints are more prone to corrosion degradation [3,4,5,6] and microstructural changes due to the heating process [3,7,8]. Takahashi et al. [9] reported that a high potential difference between the wire and the chemical compositions of the filler material results in a higher degree of deterioration. To improve wettability, the filler material often contains lead, which is toxic by nature and, therefore, dangerous to the environment and human health [10]. As silver solder is mostly used in the soldering of copper, it is necessary to also mention the high costs of silver in addition to being one of the critical raw materials [11].

Crimping, including conventional mechanical and electromagnetic crimping [12,13,14], is a simple, cost-effective, and cleaner alternative to soldering. The joint strength is generally high due to significant equilibrium pressure, ensuring the joint’s durability under various environmental conditions [15]. However, crimped connectors may face electro-chemical problems in the contact area [16], leading to higher electrical contact resistance, heating of the joints, and energy losses [12]. 

Ultrasonic welding (UW) is emerging as a promising technology for joining wires and braids without filler material. The electrical energy is converted into high-frequency mechanical vibrations of the tool, establishing a genuine metallurgical bond between the joining materials [17]. UW enables the welding of a broad spectrum of wire cross-sections using a single tool. It ensures excellent thermal and electrical conductivity and produces welds without melting the base materials [2]. It is widely recognized that the strength of ultrasonic joints is sensitive to peel strength, especially when welding wires without any additional reinforcing elements. Enhancing the peel strength of cable bundles, where the conductors of the electrical wires are interconnected, is feasible. Patents [18,19,20] indicate that incorporating reinforcing elements, such as a ring, can increase the joint’s strength, particularly if one of the electrical wires is of a smaller diameter. However, past research has been dedicated solely to the welding or strength enhancement of conductors or braids. Zhou et al. [21] also reported that increasing the welding time led to a thinning of the bonding area due to the increased penetration depth of the sonotrode, which reduced the peak tensile stress of an Al/Ti joint. Similar results were obtained by Shakil et al. [22], who showed that higher energy means a greater penetration depth in the sonotrode, which leads to weaker mechanical strength. The penetration of the sonotrode deforms and displaces the material, making it thinner and softer, which also has a negative effect on the joint strength [23].

In the operation of electric motors, one of the influential characteristics is also the effect of the joint temperature. Brisset et al. [24] reported that recrystallization and grain growth on copper wire are already noticeable at 210 °C. The study notes that nuclei grow at the expense of the deformed microstructure, enhanced by the high stored-energy difference between the nuclei and their neighbors. This is significant because recrystallization and grain growth are critical factors influencing the mechanical properties of copper wire. Wang et al. [25] reported that the tensile strength of a thin-walled copper tube decreases gradually with an increase in temperature. This is indicative of the material becoming softer and less resistant to tension as the temperature rises. Along with the decrease in tensile strength, the elongation of the copper tube initially increases but then decreases sharply due to the combined effects of grain sizes and texture components. This suggests that the ductility of the copper tube improves up to a certain point with rising temperatures before decreasing due to structural changes in the material.

This study aims to explore the impact of a thermal shock test on electrical connection and mechanical strength when ultrasonically welding three solid copper wires using a copper ring compared to a traditional method like soldering. Theoretically, the ultrasonic metal-welding process is straightforward. There are three main variables to consider: time, which represents the duration of the applied ultrasonic vibration; amplitude, indicating the longitudinal displacement of the vibration; and force, representing the compressive force applied perpendicularly to the vibration direction. The product of time, amplitude, and force equates to energy. Energy-based welding control is prevalent in most metal-welding applications, especially when the welding area is not coated with additional layers, like varnish. This control method is the most straightforward and ensures the creation of joints with consistent quality.

## 2. Materials and Methods

### 2.1. Materials and Preparation of Samples for Welding and Soldering

**Figure 1:**
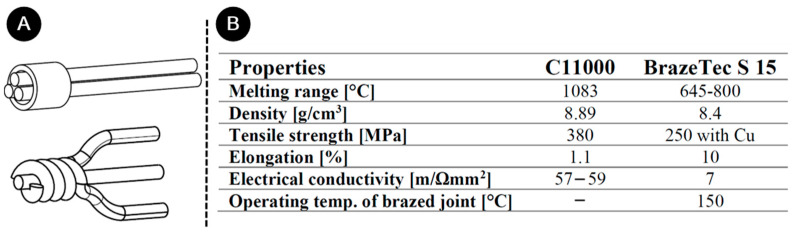
(**A**) A sketch of joint preparation for UW and soldering; (**B**) properties of copper C11000 wires [28] and BrazeTec S 15 solder [27].

A 0.75 mm diameter wire of C11000 copper coated with polyester and polyamide-imide coatings was used for experimental welding. The coating itself can withstand 200 °C [26]. Before welding, the coatings were removed using Abiofix preparation (Waldshut-Tiengen, Germany). Prior to the soldering, the copper wires were skewed (Figure 1A). BrazeTec S 15 solder was used, containing 15% Ag and 5% P [27]. The dimensions of the ring used for the UW were 2 mm at the inner diameter, 3 mm at the outer diameter, and 4 mm in length. The detailed mechanical properties of C11000 copper and BrazeTec S 15 solder can be found in Figure 1B.

### 2.2. Soldering and Ultrasonic Welding of Prepared Wires

The experimental joining of the wires was performed using soldering and ultrasonic welding. Soldering was performed with an Oswlad gas generator (Sacile, Italy) [29], producing a neutral flame with a temperature of approximately 3650 °C. We preheated the wires for 10 s and then soldered them at a temperature of ca. 450 °C. Ultrasonic welding was performed using the Branson Ultrasplice 2032S (Dietzenbach, Germany) wire welder, which converts the electric current into 20 kHz mechanical vibrations through a transducer. All joints were formed using ‘Welding To Energy’ control, which means that the main triggering parameter was the energy required to form the joint, which was kept constant for all tests, allowing the time to be adjusted to suit the condition of the materials during welding.

**Figure 2:**
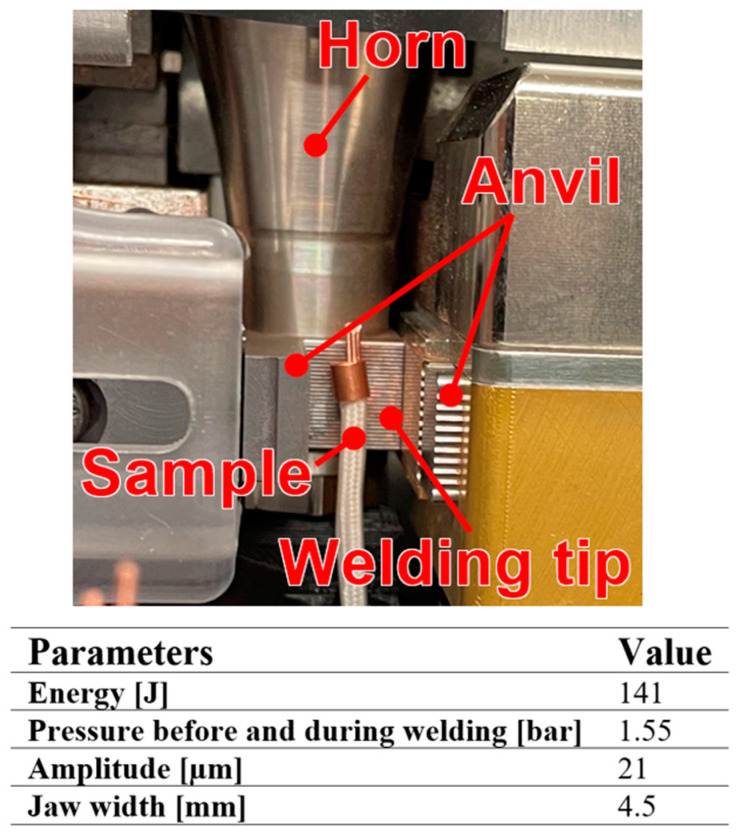
Ultrasplice 2032S device with parameters of ultrasonic welding.

All welding parameters were continuously adjusted with a closed-loop control system that monitored the accurate settings of splice width, welding energy, welding force, and amplitude. Experimental welding parameters are shown in Figure 2.

**Figure 3:**
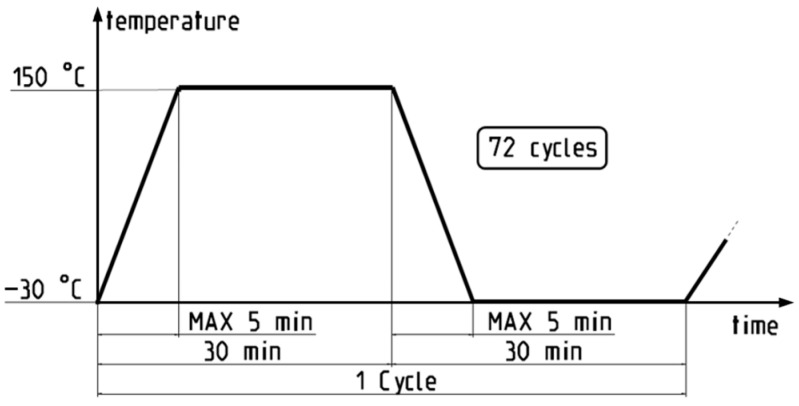
A schematic presentation of thermal shock test cycle.

The functionality of electrical and electronic components was tested in accordance with the SAE/USCAR-38 standard [30] but with an adaptation of the thermal shock test regime to align with industrial conditions. For this, 50 UW joints and 30 solder joints were subjected to alternating high and low temperatures, determined by the operating temperature range of a standard electric motor. The samples were sealed and then tested in the Espec 23-070 chamber (Düsseldorf, Germany). A schematic of the thermal shock test cycle is illustrated in Figure 3.

### 2.3. Visual Control

All samples were visually inspected after both joining methods and after the thermal shock test. During the visual inspection, we made sure that the joints had no visible cracks, that all solid wires were welded or soldered (no missing wires allowed), that the wires were not damaged (no cut or broken wires allowed), and that the joint was not burnt or over-welded (judged by visual discoloration).

### 2.4. Measurement of Electrical Resistance

**Figure 4:**
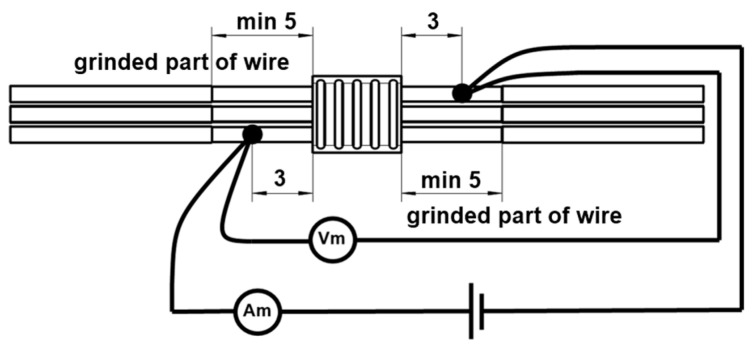
Display of contact layout for electrical resistance measurement on an example UW joint.

Electrical resistance measurements were taken from 20 samples of each UW batch and 10 samples from each soldered batch using the Keysight 34420A (Böblingen, Germany) nano-volt/micro-ohm meter. A 4-wire resistance measurement method was utilized. Before testing, the wires were grounded at the gauge attachment point. Electrical joint resistance was assessed on the outer wires, with positions indicated in Figure 4. Connection clamps on the wires were spaced 10 mm apart. Each sample underwent three measurements.

### 2.5. Analysis of Macro/Microstructure and Microhardness

Post-welding, the joints were sectioned at each joint’s midpoint and embedded in epoxy resin. The samples underwent grinding using abrasive papers of varying grits (320, 500, 800, 1200, and 4000), followed by polishing with diamond paste DP-suspension P and oxide polish with OP-S on the Struers Abramin (Ballerup, Denmark) device. The samples were then etched using ferric chloride (composed of 95 mL alcohol, 5 g FeCl_3_, and 2 mL HCl). These prepared samples, or macrosections, were inspected both pre- and post-etching using the Keyence VHX-6000 (Neu-Isenburg, Germany) optical microscope (OM) and the SEM Thermoscientific Quattro S (Waltham, MA, USA) microscope, which also verified the composition of the samples.

**Figure 5:**
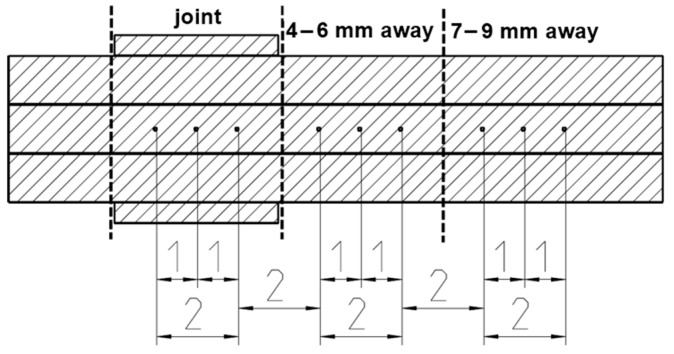
Display of the placement of microhardness measuring points on the UW joint.

Microhardness was measured using the ZHU/zwickiLine+ (Ulm, Germany) universal hardness-testing machine, following the ISO 6507 standard [31] for Vickers hardness HV 0.2, with a 200 g weight. Analysis was facilitated by the Zwick testXpert V12.3 hardness edition testing software. Two each of the UW and soldered samples, both exposed and unexposed to thermal shock, underwent testing. Figure 5 illustrates the precise measurement point locations.

In the subsequent phase, the breakage of the UW samples following tensile strength testing was also examined. The prepared samples, or macrocuts, were visually inspected using both OM and SEM to identify the typical breakage points on the wires and to describe the appearance of the breaks.

### 2.6. Mechanical Test 

**Figure 6:**
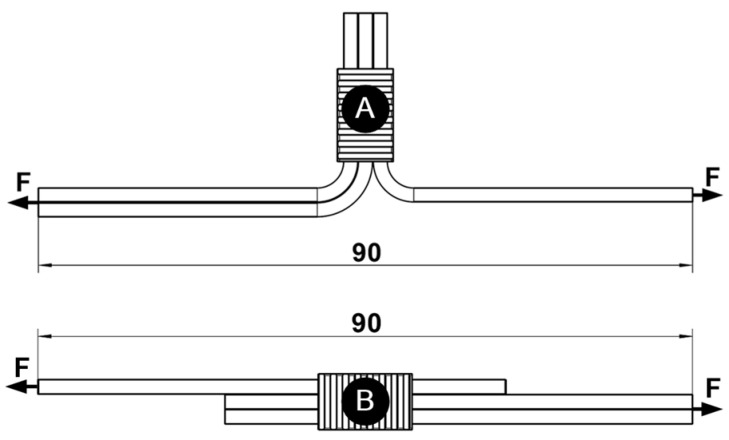
Mechanical tests: (**A**) peel-force test and (**B**) tensile strength test on the UW joint.

Tensile strength and peel-force tests were conducted in accordance with the MAN Truck & Bus AG WORKS Standard M 3455 [32]. The Zwick Z150 (Ulm, Germany) universal tensile-testing machine, equipped with the KAP-S 2 kN load cell, was utilized for both tests, and Zwick TestExpert II V 3.5 software facilitated the testing process. A pulling speed of 50 (+5) mm/min was maintained for both tests. Samples were held in grips specially designed for testing wires and cables, as illustrated in Figure 6. Peel-force testing was carried out on 26 UW samples and 20 soldered samples, as shown in Figure 6A. Tensile strength testing was executed on ten each of UW and soldered samples, as well as on wire, as depicted in Figure 6B. For each testing category, half of the samples had been exposed to thermal shock (TS), and the other half remained unexposed.

## 3. Results and Discussion

### 3.1. Preliminary Testing

**Figure 7:**
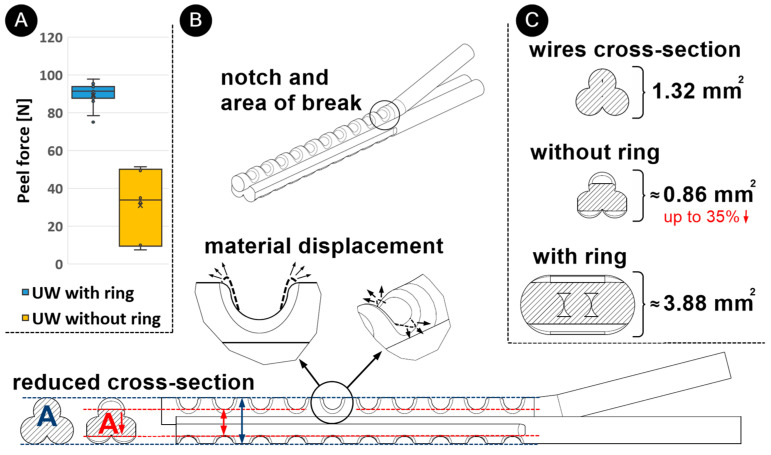
(**A**) Schematic representation of joint peel force after UW with and without a ring, (**B**) schematic representation of joint formation during UW without a ring causing material deformation and a reduction of joint cross-section (in red) and (**C**) wire cross-sections before welding and joint cross-sections after UW without and with copper ring.

Preliminary tests were carried out on the ultrasonic welding of wires with and without a ring during the optimization of the process parameters. The samples produced with optimal parameters of 141 J (with a ring) and 128 J (without a ring) were subjected to peel-force testing. The peel strength for UW samples (*n* = 6) without rings was 33.8 N ± 17.2 N. These results showed a 50% to 85% lower peel force (Figure 7A) for joints produced without a ring in comparison to the ones produced with rings. As can be seen in Figure 7B, the penetration of the sonotrode teeth into the material caused deformation and displacement of the material upon contact, which reduced the cross-section of the joint by up to 35% of the initial value (depending on the process parameters). In addition, these notches in the wire increased the notch effect, which led to a lower mechanical strength of the joint under peeling force. With ultrasonic welding with a ring, notches occurred only on the ring and had no negative effect on the joint’s mechanical strength. In addition, a twofold higher electrical resistance was found for joints without a ring. It can therefore be concluded that a ring not only improves the joint mechanically, i.e., increases the peel force, but also increases the electrical conductivity of the joint (Figure 7C) by increasing the cross-section of the joint, as the electrical current also flows through the ring. 

**Figure 8:**
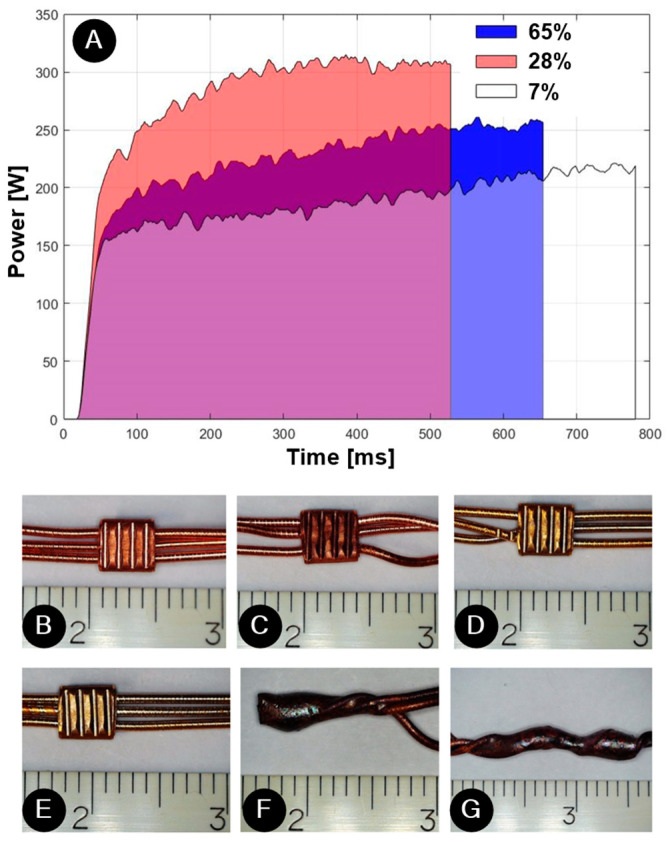
(**A**) Weld power graph: the relationship between power and time, which affects the energy and visual inspection of the samples: (**B**,**C**) UW samples not exposed to the thermal shock test, (**D**,**E**) UW samples, and (**F**,**G**) soldered sample exposed to thermal shock test.

Based on the peel-force results in the preliminary tests, we selected welding with a ring for this study. Based on the preliminary parametric analysis, we selected welding with a constant energy of 141 J. The device recorded parameters based on time and power, producing a representation of the energy expended during welding. As evident from the weld-power graph in Figure 8A, welding characteristics varied across the samples due to the coefficient of friction. This coefficient is influenced by factors such as the positions of the wires in the weld joint, surface cleanliness, heat dissipation, and more. It was observed that 28% of the samples were welded at approximately 530 ms, with a peak power of around 300 W. These samples exhibited the highest friction coefficient. The majority of samples (65%) were welded within 650 ms, with a peak power of about 250 W. In contrast, a mere 7% of the samples were welded at about 780 ms, with a peak power nearing 220 W. These latter samples had some varnish residues on the wires, resulting in a lower coefficient of friction, lower power welding, and longer welding times when welding with constant energy. Similar conclusions for less-clean welds were also reported by Kuprvs et al. [33] in their study on copper wire welding.

### 3.2. Visual Control

Examination of the samples revealed that the chosen parameters yielded high-quality welds/solders across all wires. There were no evident cracks, burns, over-welding, or damage. Figure 8B,E show that most wires at the UW joints aligned in parallel. However, as depicted in Figure 8C,D, some UW samples seemed to have wires that were partially wrapped or deformed. According to the mechanical results in Figure 7, this wrapping/deformation did not significantly impact the joints’ strength. The joints displayed in Figure 8F,G had been wrapped prior to soldering as a result of the joint preparation before the soldering process. Figure 8D,E further highlight that UW samples exposed to the thermal shock test lost their typical orange-copper hue and assumed a golden surface color. This discoloration was not seen during soldering, given that the joints already underwent high-temperature exposure during that process.

### 3.3. Results of Electrical Resistance Test

**Figure 9:**
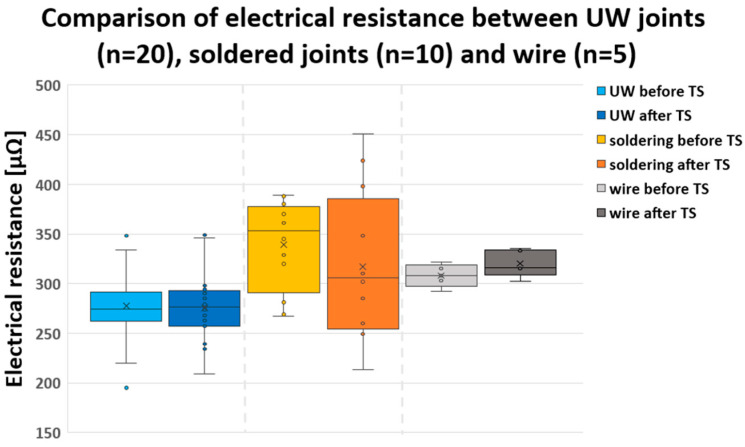
Comparison of electrical resistance between the UW joints (blue), soldered joints (orange), and wire (grey) before and after thermal shock test.

Figure 9 presents a box–whisker plot comparing the electrical resistance of UW joints, soldered joints, and the wire. For the UW samples that were unexposed to TS, the electrical resistance was 275 µΩ ± 33 µΩ, while it was 277 µΩ ± 33 µΩ for those exposed to TS. In comparison, the soldered samples that were unexposed to TS had an electrical resistance of 353 µΩ ± 44 µΩ, and it was 306 µΩ ± 70 µΩ for those exposed to TS. The inherent electrical resistance of the wires that were unexposed to TS stood at 308 µΩ ± 10 µΩ, and it was 316 µΩ ± 12 µΩ for those exposed to TS. The average values indicate that the results were closely aligned. Naturally, electrical resistances differ between distinct joining techniques and wires due to the substantial impact of the joint/wire cross-section on resistance. These results indicate that the variation in electrical resistance was 3 times less for the wire compared to UW joints and 5–7 times less than in soldered samples.

Additionally, the upper and lower quartiles were nearly identical for the UW samples. In contrast, the lower quartile exceeded the upper quartile for unexposed solder joints, while for exposed solder joints, the reverse held true. This observation, coupled with the standard deviations, suggests that human intervention plays a more substantial role in the soldering process (which was manually executed) than in UW joining. For soldering and the wire itself, exposure to TS had a marginal effect on augmenting electrical resistance. However, the thermal shock test did not influence the electrical resistance for the UW samples. This confirms that electric motor operation, simulated through a thermal shock test, does not affect the electrical integrity of the UW joint.

### 3.4. Analysis of Macro/Microstructure and Microhardness

**Figure 10:**
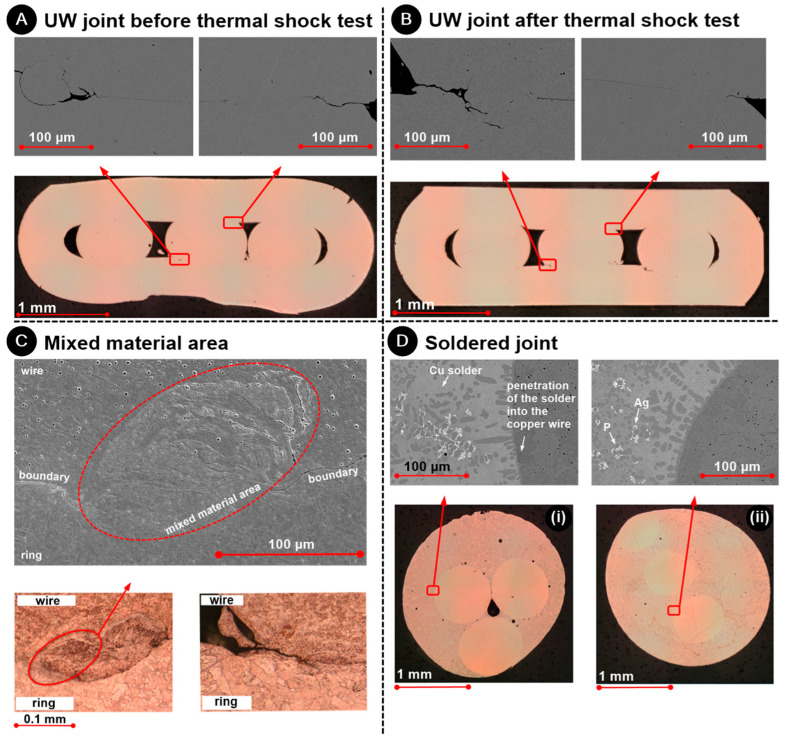
Analysis of microstructure of the UW joint (wire and ring) (**A**) before and (**B**) after the thermal shock test, (**C**) with ring–wire material mixing areas and (**D**) analysis of the microstructure with detailed views (SEM) of soldered sample (**i**) before and (**ii**) after the thermal shock test.

Both macrosection analysis and microstructure examination were employed to perform an in-depth assessment of the weld zones. Figure 10 demonstrates that for both UW samples 10A (unexposed) and 10B (exposed to the thermal shock test), there was a strong metallurgical bond between the wire and the ring; this is evidenced by the indistinguishable boundary between the wire and the ring. On the sonotrode side (A and B), there was also a notable blending of materials from both the wire and the ring. This was where the relative motion was most pronounced. Figure 10A,B depict the conditions on the side opposite the sonotrode. Here, the joint between the wire and the ring displayed a mix of areas: some were completely welded with a solid metallurgical bond, while others were not (from left to right).

Figure 10C presents a microstructural examination of the etched UW samples C (unexposed) and C (exposed to TS). Notably, there were not any major distinctions in microstructure between the two samples. The circled area further illuminates the amalgamation of the two materials. This blending caused the crystal grains of the ring to intermingle with those of the wire, providing further testament to the strong bond.

Figure 10D illustrates the microstructural study of the etched soldered samples 10D-i (exposed to TS) and 10D-ii (unexposed to TS). Again, no significant variations between the two samples were apparent. Observations showed that the solder itself had a copper base. Additionally, the solder’s penetration into the copper wire was evident. The layer of solder enveloping the wire was replete with silver dendrites. Further spectral analysis (through EDX) indicated that phosphorus tended to accumulate at the peripheries of these silver dendrite formations.

**Figure 11:**
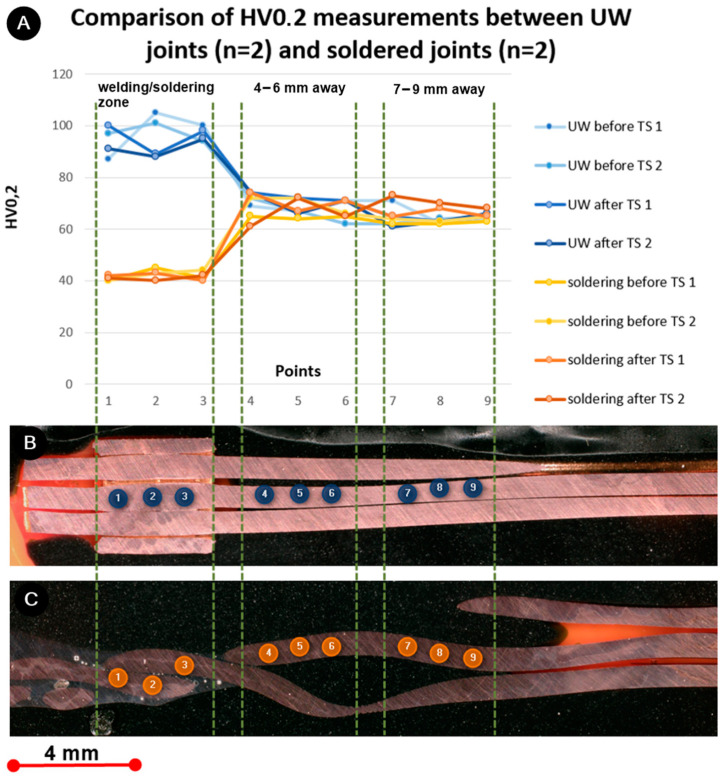
(**A**) Microhardness HV0.2 measurements of UW and soldered joints before and after thermal shock test. Positions of microhardness measurement points (1–9) for (**B**) UW joint and (**C**) soldered joint.

Figure 11 presents the microhardness measurements taken at three distinct points on the UW and soldered samples: in the center of the joint, 4–6 mm, and 7–9 mm away from the joint. For the soldered joints that were unexposed to TS, the average hardness value was 41 HV0.2 ± 2.0 HV0.2, and for those exposed to TS, it was 41 HV0.2 ± 1.1 HV0.2. The average hardness value for the UW joints that were unexposed to TS was 97 HV0.2 ± 5.7 HV0.2, and for those exposed to TS, it was 92 HV0.2 ± 4.4 HV0.2. The average hardness measurements taken 4–6 mm and 7–9 mm away from the joint ranged between 66 and 70 HV0.2 for both methods, regardless of exposure to TS. This aligns with the hardness values of the wire, validating that these zones remained unaffected by the process.

In the UW joint, the hardness value was significantly greater than in the areas 4–6 mm and 7–9 mm away from the joint, a consequence of mechanical deformation during ultrasonic welding. Conversely, the soldered joint exhibited a hardness value considerably lower than those areas, which was attributed to the elevated temperatures experienced during soldering. Notably, there was a slight reduction in hardness in the UW joint following TS (5 HV0.2), likely due to the softening process. However, no such variations were discernible for the soldered samples.

### 3.5. Mechanical Test

**Figure 12:**
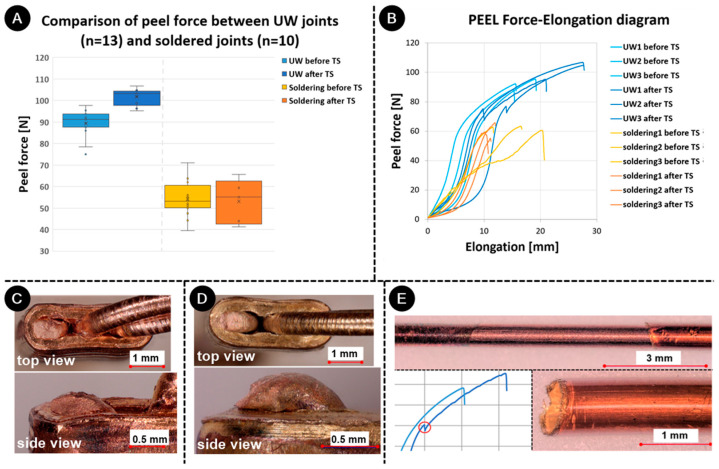
Comparison between properties of wire, UW joints, and soldered joints before and after thermal shock test: (**A**) peel force, (**B**) peel-force–elongation curves, fracture of peel-force samples (**C**) unexposed and (**D**) exposed to the thermal shock test, (**E**) the area from which the varnish peeled off and peeled varnish, with the indication (red circle) on the peel force–elongation curve.

Figure 12A displays a comparison of the peel strengths of UW and soldered joints both before and after undergoing a thermal shock test. The peel strength for UW samples that were not exposed to TS measured at 91.3 N ± 6.3 N, while for those exposed to TS, it was 103.3 N ± 3.6 N. The strength of soldered samples without exposure to TS was 53.1 N ± 7.5 N, and it was 55.1 N ± 9.3 N for samples that underwent TS. UW welding produced more robust joints than soldering. The strength of the soldered joints under peel load remained consistent before and after TS. However, an increase in strength post-TS was evident for the UW samples. The force–elongation curve (Figure 12B) indicated that elongation values also rose post-TS for the UW samples. Given different tempers, ETP copper behaves uniquely. Wires typically snap at the joint’s end, where the notch effect is most pronounced (as depicted in Figure 12C with an unexposed sample). During the peel-force test, the ring shoulders a significant portion of the load. The exposed sample (Figure 12D) revealed that the ring deformed under the wire’s force during testing, thereby diminishing the notch effect. This observation aligns with the ring’s H02 temper, suggesting that the TS-induced softening process impacted it more. A larger result, scatter for the soldering, which was evident from the standard deviation, highlights the influence of human intervention in the process, which was not a factor in UW. For the UW samples, the peel-force–elongation curve showed a force drop at approximately 70 N for those subjected to the thermal shock test. This can be attributed to the varnish peeling off the wire, especially when the varnish underwent the thermal shock test (as seen in Figure 12E). Cyclic heating and cooling during the TS test may have caused the wire and varnish to expand and contract, potentially compromising the varnish’s adhesion. Additionally, the TS test might have aged the varnish, which might have affected its elongation properties adversely. During peel or tensile tests, the wire’s further expansion and contraction may have triggered debonding. This phenomenon was not observed in soldered joints since these wires typically break at forces under 70 N before varnish peeling can occur.

**Figure 13:**
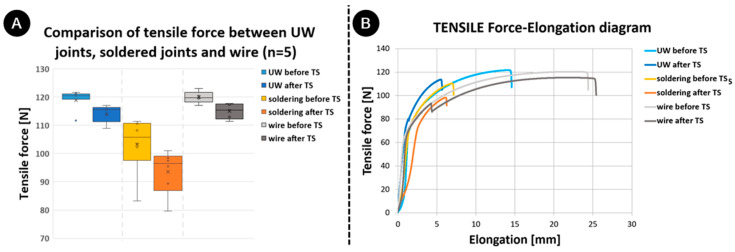
Comparison between properties of wire, UW joints, and soldered joints before and after thermal shock test: (**A**) tensile force and (**B**) tensile force–elongation curve.

Figure 13A displays the results from the tensile strength tests. The tensile strength of the UW samples that were not exposed to TS was 120.5 N ± 3.7 N, and it was 115.6 N ± 2.8 N for those exposed to TS. For soldered samples that were not exposed to TS, the tensile strength stood at 105.9 N ± 9.5 N, while it was 96.5 N ± 7.2 N for those exposed to TS. The tensile strength of wires that were not exposed to TS was 119.8 N ± 2.0 N, and it was 115.4 N ± 2.4 N for samples that were exposed to TS.

Similar to the peel test results, the UW joints proved stronger than the solder joints. The force–elongation curve (Figure 13B) indicated that the values for both tensile load and elongation decreased post-TS test for both types of joints. In the case of the wire, while the tensile load decreased, the elongation actually increased. For UW, there was a significant reduction in elongation; however, it remained within the range of the soldered samples and still maintained a higher joint strength. The solder joints also showed a larger variance in tensile strength results, which underscores the influence of workers.

The force–elongation curve for the UW samples and wire revealed a decline in force at approximately 90 N for samples that were exposed to TS. This is further evidence that this phenomenon is unaffected by the vibrations experienced at the UW. Such a phenomenon is infrequent in soldered joints, as the wires typically break at forces beneath 90 N (representing 80% of the wire’s tensile strength) before any peeling of the varnish from the wire takes place. For the soldered joints that registered higher peel or tensile forces post-TS, the same phenomena were observed.

**Figure 14:**
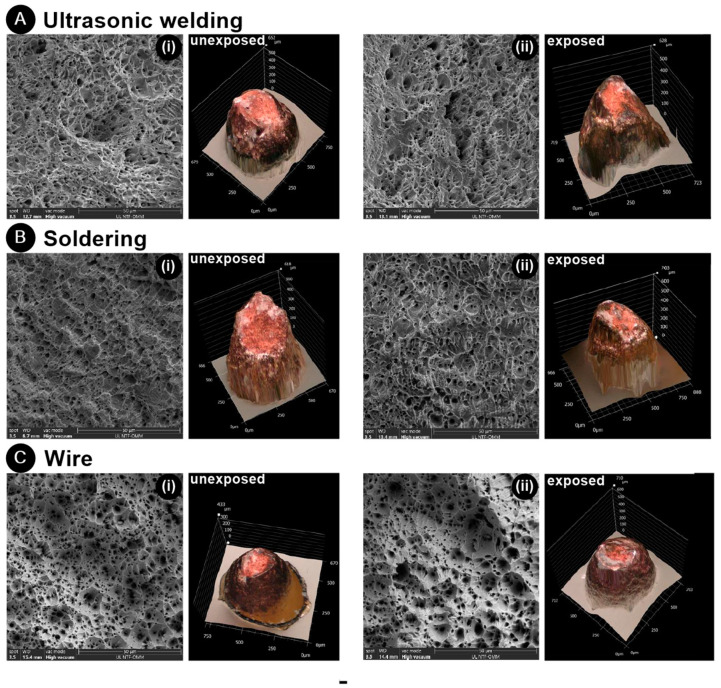
SEM images and macrographs of tensile fracture surfaces in the (**A**) UW joint, (**B**) soldered joint, and (**C**) wire ((**i**) unexposed and (**ii**) exposed to the thermal shock test).

The decrease in tensile load post-TS was further validated when examining the fractures of the specimens. In Figure 14, ductile fractures characterized by microvoid coalescence are evident in all samples. A consistent pattern in fractures A (UW) and B (soldering) was observed both before and after TS. While the fractures appeared ductile based on the force–elongation curve, the curve suggested characteristics of a brittle fracture. For UW, this discrepancy was attributed to the notch effect and the internal stresses present at the fracture. In the case of soldering, this was due to the wire thinning immediately after the joint (at the fracture) as a result of the soldering process itself. A slight difference was noted in the wire fracture in C. Larger microvoids became apparent in the wire that was exposed to TS (Figure 14(C-ii)). This observation is further substantiated by the force–elongation curve, where a greater elongation was recorded in samples that underwent TS.

## 4. Conclusions

This paper compares ultrasonic welding using a ring and the soldering of thin copper wires before and after a typical thermal shock test, which presents a simulation of an electrical motor under operation and evaluates the impact on joint properties. The assessment covered preliminary tests, visual examination, microstructure analysis, peel and tensile strength tests, and fracture analysis. The following conclusions emerged:

1. When ultrasonically welding wires without a ring, the teeth of the sonotrode cause the material to deform, reducing the cross-section by up to 35% and creating notches that weaken the mechanical strength of the joint. When ultrasonic welding with a ring, notches only occur on the ring, which maintains the mechanical strength and improves the electrical conductivity by increasing the cross-section of the joint.
2. Visual inspections suggested that both welding and soldering processes were effectively executed without any prominent issues.
3. The electrical resistances of ultrasonically welded and soldered joints, both pre- and post-thermal shock tests, as well as of the wire itself, were comparable. This indicates that the thermal shock test has minimal impact on the joint quality in each joining method. However, soldered joints displayed greater variance in electrical resistance, hinting at a stronger human influence in soldering than in semi-automated ultrasonic welding.
4. Microstructure analysis indicated a robust metallurgical bond between the wires and rings in the case of UW and between the wires and solder in soldering, regardless of exposure to thermal shock.
5. Microhardness tests revealed that during post-thermal shock, US joints softened. This effect was absent in the soldered joints. Specifically, UW joints displayed greater hardness than the wire, while soldered joints demonstrated reduced hardness compared to the wire itself.
6. Mechanical testing revealed that UW yielded more resilient joints than manual soldering. This was accompanied by more consistent outcomes, reflecting the influence of automation. Post-thermal shock, the UW method exhibited enhanced peel strength due to the ring softening and deforming under the force of the wire during testing and diminished tensile strength. In contrast, soldering saw a decline in both properties.
7. Fluctuations in plastic deformation regions were evident in the force–strain curves. These are attributed to the varnish peeling from the wire after exposure to thermal shocks. Cyclic heating and cooling during the TS test potentially weakened the varnish’s bond.
8. All fractures examined were ductile, characterized by the coalescence of microvoids. The voids on the exposed samples were larger than on the unexposed samples, demonstrating the influence of the thermal shock test on both joining methods and on the wire itself.

In summation, these findings advocate for the viability of UW joints in devices that undergo temperature fluctuations during operation.

## Data Availability

Data are contained within the article.
